# Virtual Coronary Artery Bypass Grafting

**DOI:** 10.21203/rs.3.rs-7320100/v1

**Published:** 2025-08-22

**Authors:** Wei Wu, Priyansh Patel, Parth Vikram Singh, Shijia Zhao, Yash Vardhan Trivedi, Rahul Chikatimalla, Abdulkader Shaar, Sree Sindhu Vijayarao, Varsha Miriyala, Muhammad Fiyaz Alam, Parth Munjal, Rakshita Ramesh Bhat, Kanishka Goswami, Changkye Lee, Ioanna Chatzizisi, Emmanouil S. Brilakis, George Dangas, Shahbaz Malik, Aleem Siddique, Yiannis Chatzizisis

**Affiliations:** University of Miami Miller School of Medicine; University of Miami Miller School of Medicine; University of Miami Miller School of Medicine; University of Miami Miller School of Medicine; University of Miami Miller School of Medicine; University of Miami Miller School of Medicine; University of Miami Miller School of Medicine; University of Miami Miller School of Medicine; University of Miami Miller School of Medicine; University of Miami Miller School of Medicine; University of Miami Miller School of Medicine; University of Miami Miller School of Medicine; University of Miami Miller School of Medicine; University of Miami Miller School of Medicine; University of Miami Miller School of Medicine; Minneapolis Heart Institute; Icahn School of Medicine at Mount Sinai; University of Nebraska Medical Center; University of Nebraska Medical Center; University of Miami Miller School of Medicine

**Keywords:** Coronary Artery Bypass Grafting, Fractional Flow Reserve, Computational Fluid Dynamics, Virtual Grafting

## Abstract

Coronary artery bypass grafting (CABG) offers superior long-term survival over percutaneous coronary intervention (PCI) or medical therapy in patients with complex coronary artery disease (CAD). This prospective proof-of-concept study aims to develop and validate a non-invasive computational platform that integrates coronary computed tomographic angiography (CCTA) and computational fluid dynamics (CFD) to predict post-CABG hemodynamics, including virtual grafting and fractional flow reserve (FFR) estimation. Four patients with stable multi-vessel CAD undergoing elective CABG were included. Pre-CABG CCTA was used for 3D reconstruction of coronary anatomy. Virtual bypass grafting was performed using both patient-specific graft sizes, derived from post-operative imaging and mixed-specificity graft sizes using patient-specific LIMA and standardized non-LIMA graft sizes, derived from population averages. CFD simulations were used to estimate post-CABG FFR and validated against invasive FFR measurements. Computational FFR showed strong correlation with invasive FFR (patient-specific: r^2^ = 0.92; mixed-specificity: r^2^ = 0.88). Bland-Altman analysis demonstrated minimal bias (patient-specific: 0.006 ± 0.027; mixed-specificity: −0.007 ± 0.029). Agreement with invasive FFR was 90% for patient-specific grafts (κ = 0.74, *p* = 0.016) and 80% for mixed-specificity grafts (κ = 0.41, *p* = 0.107). This virtual CABG model represents a significant advancement over existing non-invasive systems by accurately predicting post-operative hemodynamics and FFR, offering potential to optimize graft strategies and reduce reliance on invasive FFR. Future studies should explore clinical integration and large-scale validation to enhance CABG surgical planning and improve patient outcomes.

## 1. Introduction

Coronary artery bypass grafting (CABG) saves lives and remains a cornerstone in the treatment of coronary artery disease (CAD), particularly in patients with anatomically complex lesions. It has demonstrated superior long-term survival benefits compared to percutaneous coronary interventions (PCI) or medical therapy alone.[1] However, graft failure continues to pose a significant challenge—especially when grafts are placed on functionally non-significant lesions. The American College of Cardiology/American Heart Association (ACC/AHA) guidelines recommend targeting vessels with sub-occlusive stenoses when using radial artery grafts.[2] However studies found that grafts placed on non-significant lesions had a substantially higher occlusion rate (21.4%) compared to those placed on significant lesions (8.9%).[3] Another study reported a higher incidence of graft failure when bypassing non-ischemia-inducing stenoses (4.2% vs. 2.9%).[4] These findings underscore the critical role of functional assessment providing patient specific selection to optimize long-term outcomes in CABG patients while minimizing risk of complications.[5, 6]

Assessing the functional severity of coronary stenoses is performed with help of fractional flow reserve (FFR) which is defined as the ratio of maximal myocardial blood flow in the presence of stenosis to the theoretical maximal myocardial blood flow without stenosis.[7] FFR predicts the degree to which revascularization can restore optimal coronary perfusion. Despite its diagnostic value, wire-based FFR remains underutilized due to procedural risks, cost, and limited availability.[7] Angiography-derived FFR has emerged as an alternative but still necessitates invasive imaging and catheterization.[5, 8] To overcome these limitations, computational simulations have become indispensable tools for predicting the outcomes of interventional cardiovascular procedures and assessing hemodynamics noninvasively. In particular, computational fluid dynamics (CFD) combined with coronary computed tomography angiography (CTA) uses detailed anatomical data to simulate blood flow and pressure, offering a functional evaluation of coronary lesions without invasive procedures.[5, 9] Virtual CABG simulations performed with the integration of CFD with CTA provides a reliable, patient-specific framework for assessing the physiological significance of coronary stenoses virtually, thereby improving diagnostic accuracy and guiding real-time clinical decision making in the management of CAD and surgical planning for CABG.[5, 9, 10]

While the left internal mammary artery (LIMA) can often be directly visualized and modeled using coronary computed tomography angiography (CCTA), simulating grafts using other non-LIMA conduits remains a significant challenge due to the absence of preoperative imaging for these vessels. This gap in patient-specific anatomical data limits the accuracy of computational predictions and hinders precise surgical planning. To address this, our study presents a non-invasive computational framework designed to assess post-CABG hemodynamics and guide surgical strategy. The framework has two key objectives; **Aim 1**: To develop and validate patient-specific graft sizes that integrates CCTA and computational fluid dynamics (CFD) to compute fractional flow reserve (FFR) and analyze local hemodynamics; **Aim 2**: To establish a model using patient specific LIMA and fixed lumen diameters for arterial and venous non-LIMA grafts, enabling reliable hemodynamic assessment in cases where vessel imaging is unavailable. This study aims to empower surgeons with predictive insights enabling them to visualize the hemodynamic consequences of grafting decisions before entering the operating room. By leveraging non-invasive simulation, this approach supports informed decision-making for grafting in functionally non-significant lesions and helps identify patients who are most likely to benefit from invasive angiography and revascularization, ultimately improving surgical precision and patient outcomes.

## 2. Methods

### 2.1 Study design and patient selection

**Figure 1:**
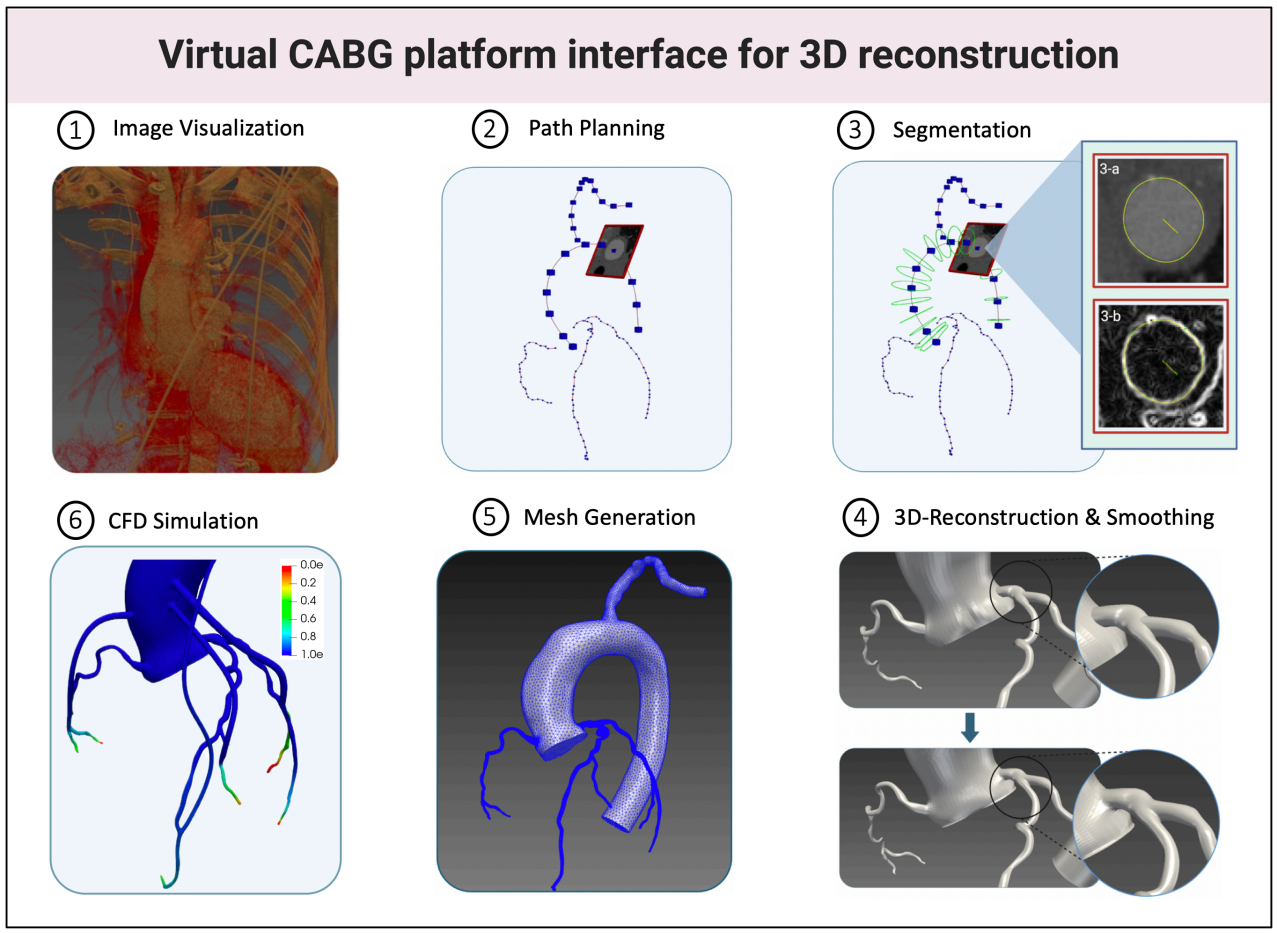
Virtual CABG platform for 3D reconstruction Illustrates the workflow for the study design; CABG: Coronary artery bypass grafting; CFD: Computational fluid dynamics

This is a prospective, proof-of-concept, single-center study. All procedures were approved by the University of Nebraska institutional review board under the IRB protocol number 0276–21-FB, and all participating patients provided informed consent. All methods were performed in accordance with the relevant guidelines and regulations. Figure 1 illustrates the workflow for the study design. A total of n = 4 participants were prospectively enrolled at the University of Nebraska Medical Center (UNMC) cardiac catheterization laboratory if they had two- or three-vessel stable coronary artery disease (CAD), defined as 50–90% stenosis as assessed by invasive coronary angiography, and were scheduled for elective CABG. Each patient underwent a pre-CABG invasive coronary angiography and CCTA scan, followed by CABG surgery with a LIMA graft to the left anterior descending artery (LAD) and additional arterial or venous grafts to the left circumflex artery (LCX) and/or right coronary artery (RCA) as required. Three months after CABG, invasive coronary angiography and FFR measurements were obtained. Patients who failed to complete post-CABG invasive angiography and FFR measurements were excluded. Table 1 summarizes the bypass characteristics of the patients, including the severity of CAD and graft configurations.

### 2.2 3D-reconstruction

**Figure 2:**
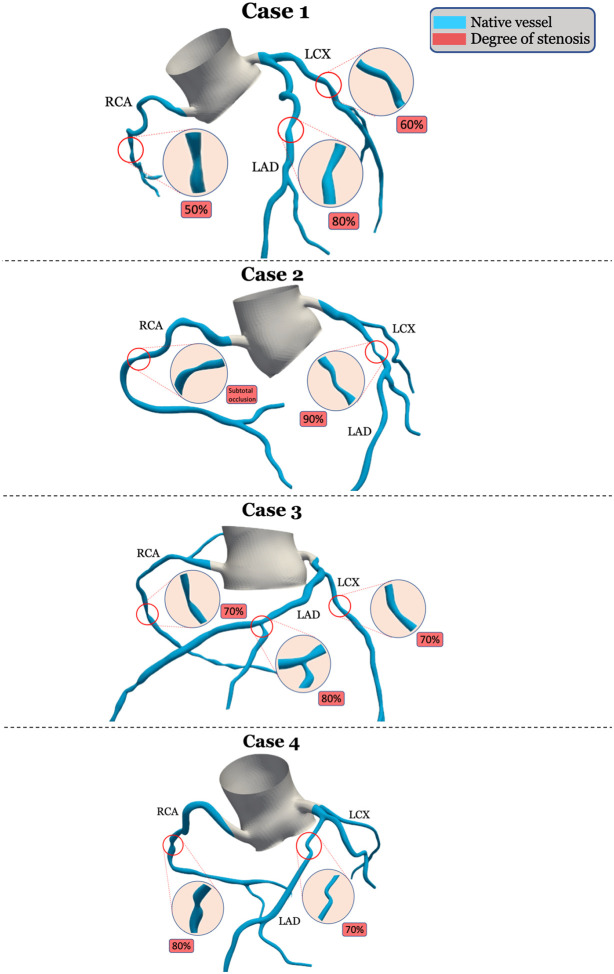
3D reconstructed stenosis in the native model Shows the pre-CABG 3D-reconstructed stenoses for all arteries; RCA: Right coronary artery; LAD: Left anterior descending artery; LCX: Left circumflex artery

Using pre-CABG CCTA images, the vascular anatomies including the aorta, left subclavian artery, right and left coronary arteries, and LIMA were 3D reconstructed using SimVascular software.[11] The 3D reconstruction process involved extracting the vessel centerline, manually segmenting the lumen, lofting 2D segmentations to create a 3D geometry, and smoothing and meshing to generate the final computational model. Figure 2 shows the pre-CABG 3D-reconstructed stenoses for all arteries.

### 2.3 Virtual CABG

**Figure 3:**
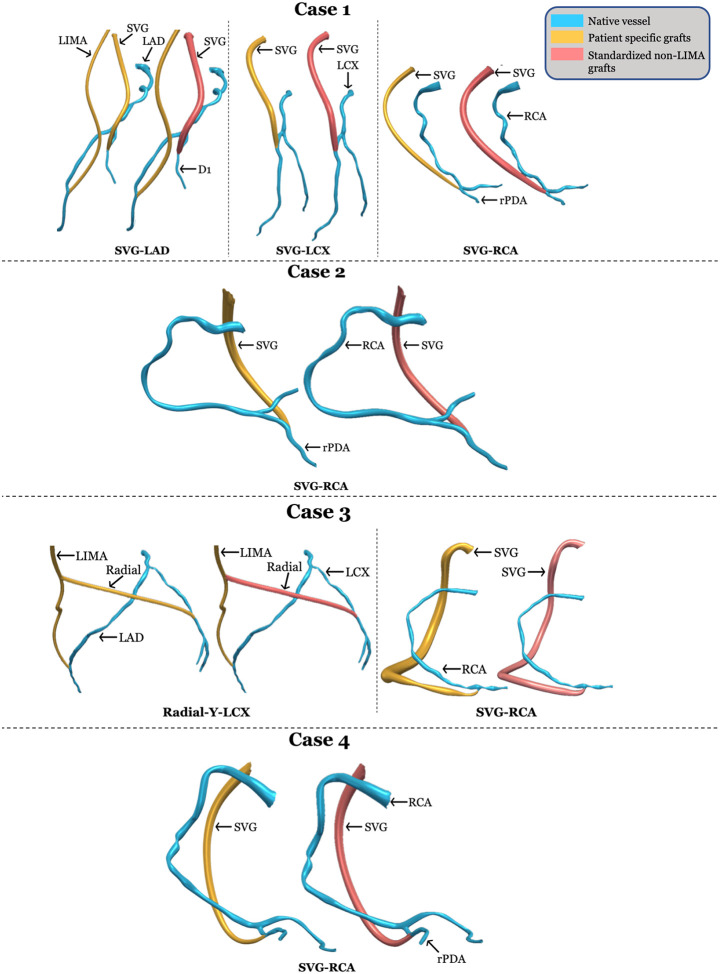
Anatomical comparison of patient specific and mixed-specificity 3D reconstruction Provides an anatomical comparison of 3D reconstruction between the patient-specific and mixed-specificity graft sizes for all cases; RCA: Right coronary artery; LAD: Left anterior descending artery; LCX: Left circumflex artery; rPDA: Right posterior descending artery; D1: Diagonal artery; SVG: Saphenous venous graft; LIMA: Left internal mammary artery

For virtual CABG, three different post-CABG models were 3D reconstructed for each case using different graft sizes. For the patient-specific graft sizes, the LIMA grafts were created using the pre-CABG CCTA while the non-LIMA grafts were created using the three months post-CABG angiograms, with the mean lumen diameters (MLD) manually measured from angiographic images to reflect actual patient anatomy. For standardized graft sizes, the MLD for both LIMA and non-LIMA grafts were assigned standardized values based on population averages keeping insertion sites of the grafts same. For simplicity, we refer to graft configurations using patient-specific LIMA and standardized non-LIMA diameters as ‘mixed-specificity’ grafts. For mixed-specificity graft sizes, as vessel imaging for LIMA is available, the LIMA grafts were created using the pre-CABG CCTA, but the MLD of non-LIMA grafts (venous and arterial) were assigned standardized values based on population averages keeping the insertion sites of the grafts same. The purpose of 3D reconstructing using standardized graft sizes is to eliminate any confounding for the mixed-specificity graft sizes. For LIMA grafts, mean diameters were used at the origin (3.05 mm), first costal cartilage (2.67 mm), fourth costal cartilage (2.22 mm) and termination (1.92 mm).[12] For saphenous vein grafts (SVGs), mean diameters of 3.9 mm for females and 4.2 mm for males were used.[13] For radial artery grafts, mean diameters of 2.27 mm for females and 2.68 mm for males were applied.[14] Fig. 3 provides an anatomical comparison of 3D reconstruction between the patient-specific and mixed-specificity graft sizes for all cases.

### 2.4 CFD setup

For each patient, CFD simulations and FFR calculations were performed under resting conditions for model tuning and hyperemic conditions for post-CABG evaluation. The CFD setup was adapted from previously validated models. The aortic inlet velocity profiles were based on 4D flow MRI data from a healthy subject.[5] Non-coronary outlets were modeled using a three-element Windkessel framework, while coronary outlets were modeled using a lumped-parameter model incorporating coronary resistance, microcirculation dynamics, myocardial compliance, and intramyocardial pressure.[15] Model tuning was performed by iteratively adjusting outlet resistance and capacitance values to maintain a normal aortic root blood pressure of 120/80 mmHg. Boundary conditions were tuned until the pressure difference between the model and target aortic pressure was within 5 mmHg. To simulate adenosine-induced hyperemia (140 mcg/kg/min, as used in invasive FFR measurements), coronary resistance was reduced to 22% and other vessel resistance to 95% of resting values.[5] Blood was modeled as an incompressible Newtonian fluid with a density of 1.06 g/cm3 and viscosity of 0.04 P. No-slip boundary conditions were applied at the vessel walls. Nine cardiac cycles were simulated, with results analyzed from the final stabilized cycle. FFR values were calculated at stenotic locations post-CABG for both the patient-specific and mixed-specificity graft sizes and compared with post-CABG invasive FFR measurements.

### 2.5 Statistical analysis

To validate the computational framework, simple linear regression analysis was performed to compare computational FFR values with invasive FFR measurements. Bland-Altman agreement analysis was conducted to assess bias between computational and invasive FFR values. Cohen’s kappa statistic was calculated indicating the degree of agreement between computational FFR and invasive FFR. The impact of standardized non-LIMA graft size on FFR accuracy was evaluated to determine whether the proposed mixed-specificity graft sizes could serve as a practical tool for preoperative CABG planning.

## 3. Results

### 3.1 Patient-specific graft size

CFD simulations were performed to calculate FFR for all patients using patient-specific 3D reconstruction. To assess the agreement between computationally derived and invasively measured FFR values, simple linear regression, Bland-Altman analysis, and Cohen’s kappa statistics were performed. Linear regression analysis demonstrated a strong correlation between computational FFR and invasive FFR measurements for patient-specific graft sizes with a regression slope of 0.99 (y = 0.99*x + 0, r^2^ = 0.92, p < 0.0001). Bland-Altman analysis further confirmed high agreement between invasive and computational FFR values for patient-specific graft sizes with a mean bias of 0.006 ± 0.027 (95% limits of agreement: −0.047 to 0.059). Kappa statistic is calculated considering an FFR threshold of 0.80—below which a stenosis is considered hemodynamically significant—the patient-specific graft sizes achieved 90% observed agreement with invasive FFR (κ = 0.74, *p* = 0.016).

### 3.2 Mixed-specificity graft sizes

**Figure 4:**
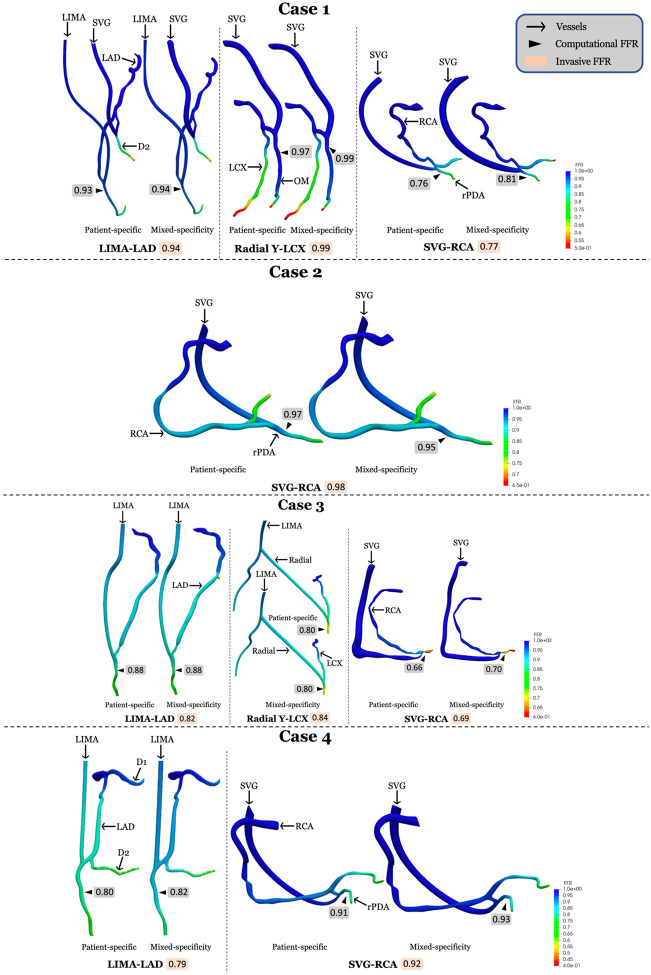
Comparative CFD analysis between patient specific and mixed-specificity graft 3D reconstruction Shows comparison between invasive FFR and computational FFR; RCA: Right coronary artery; LAD: Left anterior descending artery; LCX: Left circumflex artery; rPDA: Right posterior descending artery; D1: Diagonal artery; OM: Obtuse marginal; SVG: Saphenous venous graft; LIMA: Left internal mammary artery

CFD simulations were performed to calculate FFR for all patients using patient-specific graft size for LIMA and standardized graft size for non-LIMA grafts. As shown in Fig. 4, despite the mixed-specificity graft sizes, the computed FFR values closely matched those of the invasive FFR measurements. This indicates that mixed-specificity graft sizing does not significantly impact the overall FFR estimation. A detailed comparison of FFR values for all cases is presented in Table 2.

**Figure 5:**
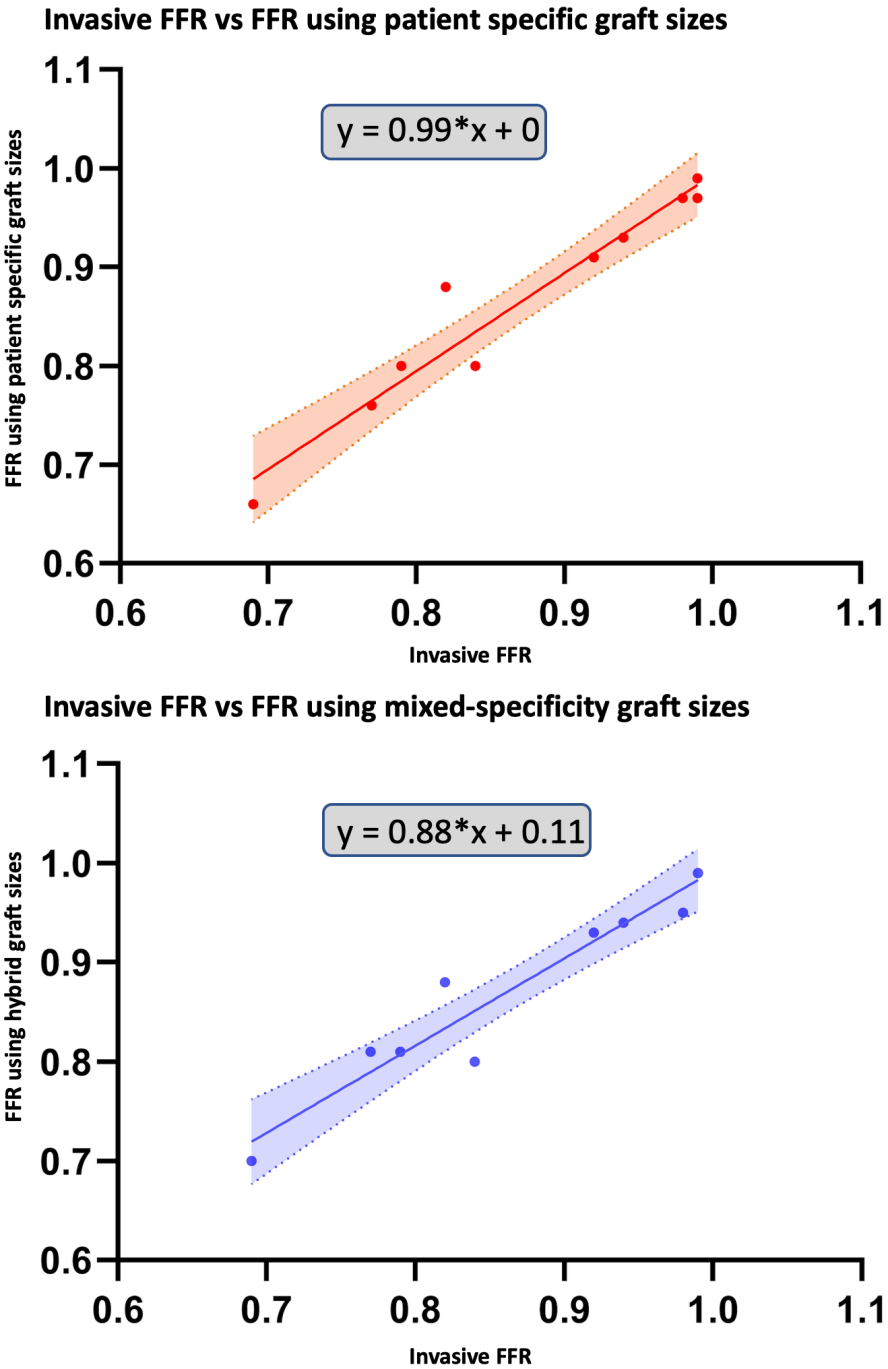
Simple linear regression curves between invasive and computational FFR Illustrates simple linear regression curves between invasive and computational FFR; FFR: Fractional flow reserve

**Figure 6:**
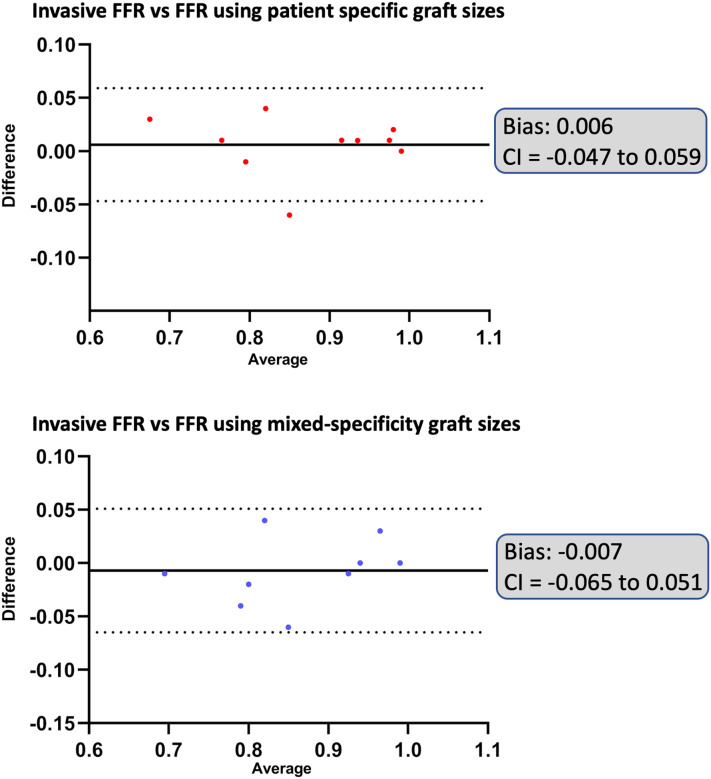
BA analysis of difference versus average between invasive and computational FFR. Illustrates BA analysis plots between invasive and computational FFR; FFR: Fractional flow reserve

Linear regression analysis demonstrated a strong correlation between computational FFR and invasive FFR measurements for mixed-specificity graft sizes with a regression slope of 0.88 (y = 0.88*x + 0.11, r^2^ = 0.88, p < 0.0001). The regression curves are illustrated in Fig. 5. Bland-Altman analysis further confirmed high agreement with a mean bias of −0.007 ± 0.029 (95% limits of agreement: −0.065 to 0.051). The corresponding Bland-Altman plots are shown in Fig. 6. Cohen’s kappa statistic is calculated considering an FFR threshold of 0.80—below which a stenosis is considered hemodynamically significant—the mixed-specificity graft size achieved 80% observed agreement with invasive FFR (κ = 0.41, *p* = 0.107).

### 3.3 Standardized graft size

CFD simulations computed FFR using standardized 3D reconstructions for LIMA and non-LIMA grafts. Linear regression showed strong correlation with invasive FFR (slope = 0.83, r^2^ = 0.93, p < 0.0001) as shown in **Supplemental Fig. 1**. Bland-Altman analysis for patient-specific graft sizes showed mean bias of 0.003 ± 0.03 (95% limits of agreement: −0.056 to 0.062) as shown in **Supplemental Fig. 2**. Using an FFR threshold of 0.80, standardized grafts had 80% agreement with invasive FFR (κ = 0.41, p = 0.107). **Supplemental Table 1** shows comparative results for invasive FFR versus computational FFR for standardized graft sizes.

## 4. Discussions

This novel study demonstrates that a virtual bypass grafting model can effectively and accurately predict postoperative hemodynamics. By leveraging a patient-specific, non-invasive computational approach, this model offers a neoteric tool for preoperative planning of bypass surgery. Specifically, it incorporates patient specific LIMA graft sizes alongside standardized diameters for non-LIMA grafts, and its predictive performance was validated against invasive fractional flow reserve (FFR) measurements.

While patient-specific graft sizing is ideal, it is not always feasible to reconstruct non-LIMA grafts, as they are not visualized preoperatively. To overcome this, we employed physiologically representative lumen dimensions to reconstruct non-LIMA grafts. Computational FFR values derived from both patient-specific and mixed-specificity graft sizes showed excellent agreement with invasive FFR measurements which is the gold standard for assessing lesion-level ischemia. The near-perfect linear correlation (slope = 0.99) for patient-specific graft sizes, and strong agreement for patient-specific LIMA and standardized non-LIMA graft sizes (slope = 0.88), provide compelling evidence that the virtual CABG approach reliably mirrors physiological reality. These findings were further supported by high concordance on Bland-Altman analysis, confirming the clinical credibility of the simulation framework.

Our findings are built upon the growing need for functionally guided CABG. Previous studies relied on post-CABG CCTA to construct native coronary artery models and computationally added stenoses based on visual angiographic assessments. This approach risked confounding the effects of the added stenoses on graft and distal LAD hemodynamics due to pre-existing native epicardial CAD.[5] By utilizing pre-CABG CCTA, our model eliminates this potential source of confounding. This focus on enhancing physiological accuracy reflects a broader trend in the literature toward functionally guided approaches to revascularization. A patient-level meta-analysis study demonstrates significantly higher graft patency when bypasses were directed toward FFR-positive lesions.[16] Another study consolidates the prognostic value of CT-derived FFR in predicting graft patency and post-operative outcomes, even in retrospective multicenter settings.[17] Table 3 shows the comparative analysis of current literature which confirms that physiology along with anatomy should be the cornerstone of graft selection.[2, 16–20] Our study translates this principle into practice by providing a reliable, noninvasive tool to implement it in the surgical workflow.

The clinical importance of this approach is further consolidated by the FAME 3 trial. In patients with three-vessel disease, FFR-guided PCI was compared with CABG. While CABG maintained its superiority in terms of long-term event reduction, it came at the cost of higher perioperative complications that were observed across the patient spectrum.[19, 21, 22] These findings reaffirm the need to optimize surgical planning. Our framework directly addresses this by enhancing preoperative planning with precise functional guidance. Even the mixed-specificity graft sizes correlated strongly with invasive FFR, demonstrating their value as a practical and accurate tool in real-world settings.

The model is consistent with the latest ACC Guidelines on coronary revascularization, which advocate for the use of fractional flow reserve (FFR) to guide bypass graft selection—particularly when considering arterial conduits such as the radial artery.[2] This computational framework also aligns with the evolving paradigm of multidisciplinary, data-driven cardiovascular care. As revascularization decisions increasingly rely on collaborative input, virtual simulations offer an objective tool to assess procedural risk and visualize the physiological consequences of different surgical strategies prior to operative intervention.[23, 24]

Our study presents strong evidence that CCTA-based computational modeling of CABG is clinically accurate. By combining high-resolution imaging with validated flow simulation, we offer a framework that improves how surgeons can select, plan, and execute coronary bypass surgery. Our study provides evidence that computational fluid dynamics offers a reliable, non-invasive alternative to FFR for CABG planning. This work also sets the stage for broader surgical planning in modern cardiovascular medicine. The ability to simulate noninvasively coronary hemodynamics using readily available imaging and predicting outcomes with this level of accuracy transforms CABG into a functionally informed and precise procedure. Thus, validating its role in optimizing surgical strategy and minimizing complications in the most definitive treatment of coronary artery disease.

## 5. Limitations

Though these findings are promising, the small sample size of this study (n = 4) limits its generalizability. Our study is serving as a proof-of-concept for future studies with larger and more diverse cohorts that can validate these findings further and improve the computational model. Moreover, the current platform is computationally intensive and requires significant expertise that may preclude implementation in routine clinical practice. To ensure widespread adoption, facilitating smooth integration of the platform in already existing imaging workflows will be essential. Additional studies should look at the platform’s ability to forecast long-term clinical outcomes (e.g., graft patency and patient survival), and evaluate its potential applicability to other vascular surgical procedures. Novel imaging modalities with state-of-the-art machine learning algorithms could be adopted to further optimize the predictive power and operational efficiency of the model.

## 6. Conclusion

Current FFR-CT platforms are limited in that they do not provide FFR estimates for bypass grafts. The ability of our framework to compute FFR values within grafts represents a significant advancement over existing non-invasive systems. This novel study addresses the key limitation of unavailable preoperative imaging for non-LIMA grafts in CABG planning. It validates a non-invasive, CCTA-based CFD framework that accurately predicts post-CABG hemodynamics, showing strong agreement with invasive FFR. Both patient-specific and mixed-specificity graft sizing yielded FFR estimates comparable to invasive measurements, supporting its clinical utility for surgical planning.

## Supplementary Material

Supplementary Files

This is a list of supplementary files associated with this preprint. Click to download.

- SupplementMaterial.pdf

## Figures and Tables

**Table 1:** Baseline disease characteristics for eligible patients.

Case No.	Age	Sex	Artery involved	Severity of CAD	Grafts
1	63	F	LAD	Proximal LAD: 80% Mid LAD: 70% Distal LAD: 80%	LIMA-LAD SVG-D_2_
			LCX	Proximal OM1: 60%	SVG-OM
			RCA	Proximal RCA: 50% Distal RCA: 80%	SVG-rPDA
2	72	M	LAD	Mid LAD: 90% calcific	LIMA-LAD
			RCA	Mid RCA with heavily calcified subtotal occlusion with TIMI-3 flow distally	SVG-rPDA
3	58	M	LAD	Mid LAD: 80% with in-stent restenosis Distal LAD: 80% D1: 70%	LIMA-LAD
			LCX	LCX: 70%	Radial Y-OM_1_
			RCA	RCA: 70%	SVG-RCA
4	68	M	LAD	Mid LAD: 70% Distal LAD: 70%	LIMA-LAD
			LCX	Mid LCX: 80%	-
			RCA	Mid RCA: 80%	SVG-PDA with sequential to acute marginal branch

RCA: Right coronary artery; LAD: Left anterior descending artery; LCX: Left circumflex artery; rPDA: Right posterior descending artery; D: Diagonal artery; OM: Obtuse marginal; SVG: Saphenous venous graft; LIMA: Left internal mammary artery

**Table 2:** Computational versus invasive FFR results

Case No.	Vessel	FFR (Invasive)	FFR (Patient-specific graft size)	%Error	FFR (Mixed-specificity graft size)	%Error
1	LAD	0.94	0.93	−1.06	0.94	0.00
	RCA	0.77	0.76	−1.30	0.81	5.19
	OM1	0.99	0.97	−2.02	0.99	0.00
2	LCX	0.99	0.99	0.00	0.99	0.00
	RCA	0.98	0.97	−1.02	0.95	−3.06
3	LAD	0.82	0.88	7.32	0.88	7.32
	LCX	0.84	0.8	−4.76	0.8	−4.76
	RCA	0.69	0.66	−4.35	0.7	1.45
4	LAD	0.79	0.8	1.27	0.82	3.80
	RCA	0.92	0.91	−1.09	0.93	1.09

FFR: Fractional flow reserve

**Table 3:** Comparative analysis of current literature

Study	Objective	Key finding	Conclusion
Zu et al.^17^	To determine the prognostic value of CT-derived FFR in patients undergoing CABG.	CT-FFR independently predicted graft patency and major adverse cardiovascular outcomes, demonstrating its clinical utility.	CT-derived FFR is a reliable noninvasive surrogate for functional assessment and can guide optimal graft target selection in surgical planning.
Lawton et al.^2^	To provide updated, evidence-based recommendations for coronary artery revascularization.	Emphasized the utility of FFR in surgical decision-making, particularly for selecting targets when using arterial conduits such as the radial artery.	FFR should be considered in preoperative planning for CABG to improve the long-term effectiveness of surgical revascularization.
Toth et al.^16^	To assess the effect of FFR-guided lesion selection on arterial graft patency.	Grafts placed on FFR-positive lesions showed significantly higher long-term patency rates than those on functionally non-significant lesions.	Functional guidance using FFR significantly improves graft outcomes, supporting its integration into CABG planning strategies.
Jayakumar et al.^18^	To evaluate the impact of FFR-based preoperative planning on arterial graft function and clinical outcomes.	Functional assessment using FFR led to better graft function and reduced early graft occlusion, though the findings were observational.	Incorporating preoperative physiological data into surgical planning may enhance graft durability and should be considered in routine CABG workflows.
Fearon et al.^19^	To compare FFR-guided PCI with CABG in patients with three-vessel disease.	CABG demonstrated superior long-term outcomes but was associated with higher perioperative complication rates, which were consistent across all patient subgroups.	The consistent perioperative risk profile of CABG underscores the need for optimized surgical planning. FFR-guided strategies may help mitigate unnecessary grafting and improve procedural safety.
Glineur et al.^20^	To evaluate the impact of preoperative fractional flow reserve (FFR) on arterial graft function following CABG.	Grafts anastomosed to lesions with FFR > 0.80 (non-significant) were associated with impaired anastomotic flow and increased risk of graft failure.	Preoperative FFR strongly predicts post-operative arterial graft performance. Bypassing non-ischemia-producing lesions may compromise long-term patency.

## Data Availability

All data generated or analysed during this study are included in this published article (and its Supplementary Information files).

## Abbreviations

CAD: Coronary artery disease
CABG: Coronary artery bypass grafting
CCTA: Coronary computed tomographic angiography
CFD: Computational fluid dynamics
FFR: Fractional flow reserve
LIMA: Left internal mammary artery
MLD: Mean lumen diameters
OM: Obtuse marginal artery
PCI: Percutaneous coronary interventions
SVG: Saphenous vein graft
